# Latent Profiles of Peer Attachment and Their Longitudinal Associations with Cyberbullying Perpetration and Cybervictimization Among Chinese Adolescents

**DOI:** 10.3390/bs16101852

**Published:** 2026-10-09

**Authors:** Min Xu, Xiaohui Xu, Yaohua Zhang, Song Chang, Yunyun Huang, Sufei Xin

**Affiliations:** 1 College of Education, Ludong University, Yantai 264025, China; xumin_psy@ldu.edu.cn (M.X.); xuxh3273@ldu.edu.cn (X.X.); zhangyh@ldu.edu.cn (Y.Z.); songchang@ldu.edu.cn (S.C.); huangyy2011@ldu.edu.cn (Y.H.); 2 Collaborative Innovation Center for the Mental Health of Youth from the Era of Conversion of New and Old Kinetic Energy Along the Yellow River Basin, Yantai 264025, China; 3 Provincial Experimental Teaching Demonstration Center of Psychology, Yantai 264025, China

**Keywords:** peer attachment, cyberbullying perpetration, cybervictimization, latent profile analysis

## Abstract

As online interpersonal communication has become increasingly common in adolescents’ daily lives, cyberbullying perpetration and cybervictimization have emerged as important concerns. Although peer attachment may be relevant to cyberbullying involvement, previous research has predominantly adopted variable-centered approaches, with limited attention to heterogeneity in peer attachment. Using a person-centered approach, this study aimed to identify latent profiles of peer attachment among adolescents and examine whether peer attachment profiles were prospectively associated with cyberbullying perpetration and cybervictimization assessed six months later. A total of 1851 Chinese adolescents (845 boys and 1006 girls; aged 11–17 years, *M* = 13.53, *SD* = 1.45) participated in two assessments conducted six months apart. Peer attachment was assessed at Time 1, whereas cyberbullying perpetration and cybervictimization were assessed at Time 2. Latent profile analysis (LPA) identified four profiles: secure profile (22.04%), intermediate profile (36.63%), low attachment profile (32.74%), and alienated profile (8.59%). Similar four-profile solutions emerged independently among boys and girls. Cyberbullying perpetration and cybervictimization differed significantly across profiles. The secure profile showed the lowest levels of both outcomes, followed by the intermediate profile, whereas the low attachment and alienated profiles showed the highest levels, with no significant differences between these two profiles on either outcome. These findings highlight the potential value of considering heterogeneity in peer attachment when identifying adolescents who may differ in their levels of subsequent cyberbullying involvement.

## 1. Introduction

Cyberbullying has become increasingly prevalent among adolescents and has emerged as a major public health concern worldwide. Cyberbullying refers to any behavior performed through electronic or digital media by individuals or groups that repeatedly communicates hostile or aggressive messages intended to inflict harm or discomfort on others (Tokunaga, 2010). Cyberbullying is characterized by anonymity, invisibility, and the potential to occur at any time and in any place (Slonje et al., 2013; Zhu et al., 2021). These features may make it difficult for victims to escape negative online interactions, thereby exposing them to substantial psychological harm (Tokunaga, 2010; Van Geel et al., 2014). A growing body of research indicates that adolescence is a developmental period marked by a relatively high prevalence of cyberbullying, and Chinese adolescents likewise face a considerable risk of cyberbullying involvement (J. K. Chen & Chen, 2020; J. Li et al., 2019; J. Xing et al., 2023). Cyberbullying may have a wide range of adverse consequences for adolescents across different involvement roles. Research has shown that cybervictimization is closely associated with mental health problems, including depression, anxiety, and suicide risk, whereas cyberbullying perpetration is associated with externalizing problems and poorer psychosocial adjustment (Kasturiratna et al., 2025; Marciano et al., 2020; Marengo et al., 2026; Tabares et al., 2024; J. Zhou et al., 2023). Moreover, cyberbullying involvement appears to show some continuity over time (Camacho et al., 2023; Marciano et al., 2020). Importantly, cyberbullying perpetration and cybervictimization should not be viewed as entirely independent forms of cyberbullying involvement. Empirical studies have documented a close association and frequent co-occurrence between them (Güllü et al., 2023; Kowalski et al., 2014; Marciano et al., 2020; Yildiz Durak & Saritepeci, 2020). Adolescents may be involved in cyberbullying as both perpetrators and victims, and the two forms of involvement may share common correlates (Jiang et al., 2024). Accordingly, examining factors associated with both cyberbullying perpetration and cybervictimization is important for understanding their occurrence and informing effective prevention and intervention strategies.

In recent years, researchers have increasingly examined multiple factors associated with adolescents’ cyberbullying perpetration and cybervictimization. Previous studies have identified a range of risk factors, including online activity, aggressive cognition, traditional bullying, internalizing problems, antisocial personality traits, neuroticism, and depressive symptoms, all of which have been associated with a greater likelihood of cyberbullying perpetration or cybervictimization among adolescents (Guo, 2016; D. Zhang et al., 2020). However, existing research has focused predominantly on factors associated with greater cyberbullying involvement, with comparatively less attention paid to individual or contextual resources associated with lower levels of involvement. A limited number of studies have found that individual psychological resources, such as a growth mindset, cognitive empathy, and emotional intelligence (Calvete et al., 2023; C. Lee & Shin, 2017; Zhu et al., 2021), as well as school-level factors, such as a positive school climate and effective school supervision (Kasturiratna et al., 2025), are associated with a lower risk of cyberbullying perpetration or cybervictimization among adolescents. Relatively little research, however, has examined cyberbullying involvement from the perspective of adolescents’ offline interpersonal relationships. Because cyberbullying occurs within interpersonal contexts, the trust, sense of security, and interpersonal skills associated with adolescents’ offline relationships may also be relevant to how they interact with others online. It is therefore important to further examine relational resources embedded in adolescents’ offline peer relationships that are associated with lower levels of cyberbullying involvement.

Attachment refers to an emotional bond that develops early in life based on how primary caregivers respond to an individual’s needs (Bowlby, 1982). As individuals enter adolescence, they spend less time with parents or caregivers and more time interacting with peers (J. Y. Lee & Park, 2017), who increasingly become important sources of social support (Gorrese & Ruggieri, 2012). Accordingly, attachment relationships during adolescence may extend beyond parents to include peers. As an important aspect of adolescents’ interpersonal relationships, peer attachment is closely associated with their psychosocial adjustment (Wright, 2025; Z. Xing & Ge, 2025). Existing research provides preliminary evidence that peer attachment may be associated with lower levels of cyberbullying involvement. With respect to cyberbullying perpetration, some evidence has directly linked peer attachment to cyberbullying perpetration. For example, a study of Indonesian adolescents found that secure peer attachment was negatively associated with cyberbullying perpetration, whereas insecure peer attachment was positively associated with it (Riany & Utami, 2025). Evidence regarding cybervictimization is more limited, but studies of related peer resources suggest that supportive peer relationships may be associated with lower levels of cybervictimization. For example, friend support has been found to be negatively associated with cybervictimization among adolescents (Arató et al., 2022). Taken together, these findings suggest that positive peer relationship resources may be associated with lower levels of cyberbullying involvement. Compared with general peer relationship resources, peer attachment captures a closer and more emotionally significant relational bond characterized by trust, communication, and emotional connectedness, and may therefore be particularly relevant to understanding both cyberbullying perpetration and cybervictimization.

However, previous research has predominantly adopted a variable-centered approach to examine the associations of peer attachment with cyberbullying perpetration and cybervictimization. Although variable-centered research is useful for identifying overall associations between peer attachment and these two forms of cyberbullying involvement, it generally assumes that individuals within a sample are drawn from a relatively homogeneous population (Howard & Hoffman, 2018). This approach may therefore overlook heterogeneity in adolescents’ peer attachment and differences in cyberbullying perpetration and cybervictimization across distinct patterns of peer attachment, thereby limiting a nuanced understanding of these associations. A person-centered approach is therefore well suited to identifying latent profiles of peer attachment among adolescents. Existing person-centered studies have primarily focused on configurations of attachment relationships with different attachment figures (Flykt et al., 2021; Gao et al., 2024; He et al., 2018), whereas relatively few studies have examined the latent structure of adolescent peer attachment itself. One previous study used K-means cluster analysis to identify four clusters of peer attachment among adolescents (X. Q. Li et al., 2009), providing preliminary evidence of heterogeneity in peer attachment. However, traditional cluster analysis has limitations in evaluating model fit and accounting for uncertainty in classification. Unlike traditional cluster analysis, latent profile analysis (LPA) is a model-based person-centered approach that allows alternative profile solutions to be compared using model fit information and estimates the probability that each individual belongs to a given profile (Spurk et al., 2020).

LPA therefore offers a model-based approach to identifying latent profiles of adolescent peer attachment. Using a two-wave prospective design, the present study examined whether peer attachment profiles assessed at Time 1 were associated with cyberbullying perpetration and cybervictimization assessed six months later. Because these outcomes were not assessed at Time 1, the study examined differences in their subsequent levels rather than changes over time. The findings may contribute to understanding the prospective associations between peer attachment configurations and later cyberbullying involvement and may inform future research on profile-informed assessment and prevention.

Accordingly, the present study addressed the following research questions:

RQ1: What empirical profiles of peer attachment can be identified on the basis of peer trust, peer communication, and peer alienation?

RQ2: Are the empirically derived peer-attachment profiles prospectively associated with cyberbullying perpetration and cybervictimization assessed six months later?

Because the number and characteristics of the profiles were not specified a priori, the profile-identification analysis was exploratory.

## 2. Materials and Methods

### 2.1. Participants

Participants were recruited from six secondary schools in Shandong Province, China, using convenience sampling. Data were collected at two time points approximately 6 months apart. Of the 2028 adolescents who participated at baseline, 1851 (91.3%) were retained at follow-up, whereas 177 (8.7%) were lost to follow-up because of school transfers, leaves of absence, illness, or other reasons. The final analytic sample therefore comprised 1851 adolescents who participated in both waves and provided sufficient data for inclusion in the analyses. Of these participants, 845 (45.65%) were boys and 1006 (54.35%) were girls. Participants ranged in age from 11 to 17 years (*M* = 13.53, *SD* = 1.45). The sample included 283 Grade 7 students (15.29%), 781 Grade 8 students (42.19%), 518 Grade 9 students (27.98%), 46 Grade 10 students (2.49%), and 223 Grade 11 students (12.05%). Regarding paternal education, 1105 fathers (59.70%) had a junior high school education or less, 498 (26.90%) had a senior high school education, 132 (7.13%) had a junior college education, and 116 (6.27%) had a bachelor’s degree or higher. Regarding maternal education, 1206 mothers (65.15%) had a junior high school education or less, 408 (22.04%) had a senior high school education, 134 (7.24%) had a junior college education, and 103 (5.56%) had a bachelor’s degree or higher.

To examine whether attrition was systematic, participants retained at follow-up were compared with those lost to follow-up on baseline demographic characteristics and peer-attachment variables. Participants lost to follow-up were significantly older (*M* = 14.11, *SD* = 1.33) than those retained (*M* = 13.53, *SD* = 1.45), *t*(2026) = 5.09, *p* < 0.001, Cohen’s *d* = 0.40. The grade distribution also differed significantly between the two groups, χ^2^(4) = 81.93, *p* < 0.001, Cramér’s *V* = 0.20. However, no significant group differences were found in sex, χ^2^(1) = 2.60, *p* = 0.107, φ = 0.04; paternal education, χ^2^(3) = 0.61, *p* = 0.894, Cramér’s *V* = 0.02; or maternal education, χ^2^(3) = 6.75, *p* = 0.080, Cramér’s *V* = 0.06. Participants retained at follow-up and those lost to follow-up did not differ significantly in peer trust, Welch’s *t*(248.43) = 1.12, *p* = 0.265, Cohen’s *d* = 0.07; peer communication, Welch’s *t*(284.96) = −0.10, *p* = 0.919, Cohen’s *d* = −0.01; or peer alienation, *t*(2026) = −1.81, *p* = 0.070, Cohen’s *d* = −0.14. These findings indicate that attrition was associated with age and grade but not with sex, parental education, or the focal peer-attachment variables.

### 2.2. Measures

Peer Attachment. Peer attachment was assessed using the peer attachment subscale of the Inventory of Parent and Peer Attachment (IPPA; Armsden & Greenberg, 1987). The subscale assesses three dimensions: peer trust, peer communication, and peer alienation. Peer trust reflects mutual trust and respect for each other’s needs and wishes (e.g., “My friends understand me”). Peer communication refers to the perceived quality of involvement, responsiveness, and verbal communication (e.g., “I can tell my friends about my problems and concerns”). Peer alienation reflects feelings of anger, isolation, and separation from peers (e.g., “I feel angry with my friends”; Armsden & Greenberg, 1987). The peer attachment subscale consists of 25 items rated on a 5-point scale ranging from 1 (never) to 5 (always). Higher scores on peer trust and peer communication reflect greater trust and communication, whereas higher scores on peer alienation reflect greater alienation from peers. A confirmatory factor analysis was conducted to examine the factor structure of the peer attachment subscale. Items were treated as approximately continuous indicators, and the model was estimated using robust maximum likelihood estimation (MLR). Item 5 showed an essentially zero and nonsignificant standardized factor loading (λ = −0.02, *p* = 0.389), whereas Item 9 showed a significant negative loading on peer alienation (λ = −0.21, *p* < 0.001), opposite to the theoretically expected direction. Given these anomalous factor loadings, both items were removed as a data-informed modification, consistent with general methodological recommendations for item evaluation and scale refinement (Clark & Watson, 2019). Their removal was further supported by a previous validation of the Chinese IPPA-R, which reported similarly weak performance for these items and potential semantic ambiguity for Item 9 (Y. L. Zhang et al., 2011). The final measure used in the present study therefore contained 23 items. The revised three-factor model was estimated without correlated item residuals or cross-loadings. The model showed generally acceptable fit: χ^2^/df = 6.15, CFI = 0.927, TLI = 0.918, RMSEA = 0.053, 90% CI [0.050, 0.055], and SRMR = 0.035. All STDYX-standardized factor loadings were statistically significant (*ps* < 0.001) and ranged from 0.476 to 0.840. No further items were removed. Cronbach’s α was 0.87 for the overall peer attachment subscale and 0.92, 0.91, and 0.76 for peer trust, peer communication, and peer alienation, respectively.

Cyberbullying Perpetration and Cybervictimization. Cyberbullying perpetration and cybervictimization were assessed using the Revised Cyber Bullying Inventory (RCBI; Topcu & Erdur-Baker, 2010), which has previously been used and supported in research with Chinese adolescents (Chu & Fan, 2017). The RCBI consists of two parallel 14-item subscales assessing cyberbullying perpetration (e.g., “Posting false photos or information online to defame someone”) and cybervictimization (e.g., “Someone posted false photos or information online to defame me”). Items are rated on a 4-point scale ranging from 1 (never) to 4 (more than 3 times), with higher scores indicating a higher frequency of cyberbullying perpetration or cybervictimization. Confirmatory factor analyses were conducted separately for the cyberbullying perpetration and cybervictimization subscales. Items were treated as ordered categorical indicators, and the models were estimated using weighted least squares with mean and variance adjustment (WLSMV). The one-factor model for cyberbullying perpetration showed good fit to the data, χ^2^/df = 2.28, CFI = 0.99, TLI = 0.99, RMSEA = 0.026, 90% CI [0.021, 0.031], SRMR = 0.05. Standardized factor loadings ranged from 0.68 to 0.92, and all were statistically significant, *ps* < 0.001. No items were removed, and no cross-loadings or correlated item residuals were specified for this subscale. The one-factor model for cybervictimization showed acceptable but comparatively weaker fit, χ^2^/df = 13.02, CFI = 0.93, TLI = 0.92, RMSEA = 0.08, 90% CI [0.08, 0.09], SRMR = 0.08. Inspection of the modification indices and item content suggested local dependence between Items 1 and 2. Both items concern unauthorized access to or theft of personal digital information or online accounts. On this substantive and empirical basis, one residual covariance between Items 1 and 2 was freely estimated. This data-informed modification resulted in good model fit, χ^2^/df = 4.96, CFI = 0.98, TLI = 0.97, RMSEA = 0.05, 90% CI [0.04, 0.05], and SRMR = 0.05. All STDYX-standardized factor loadings were statistically significant (*ps* < 0.001) and ranged from 0.59 to 0.90. In the present study, Cronbach’s α coefficients were 0.82 for cyberbullying perpetration and 0.88 for cybervictimization.

### 2.3. Research Procedure

The study protocol was approved by the Institutional Review Board (IRB) of Ludong University. Before the study began, all students and their guardians were informed that participation was entirely voluntary and that they had the right to withdraw at any time. Informed consent was obtained from all participants and their guardians at each wave of data collection. The study employed a two-wave prospective design. The first assessment was conducted in late November 2021 (Time 1; T1), at which time peer attachment was assessed. Paper-and-pencil questionnaires were administered in person by master’s students serving as trained research assistants. Before questionnaire administration, the research assistants read standardized instructions to the participants and emphasized that participants’ responses and study data would be kept confidential. Completed questionnaires were collected on site by the same research assistants. The second assessment was conducted six months later, in late May 2022 (Time 2; T2), at which time cyberbullying perpetration and cybervictimization were assessed. The data collection procedures at T2 were the same as those used at T1.

### 2.4. Data Analysis

SPSS 26.0 was used for data management and statistical analyses, including the assessment of common method bias, correlation analysis, and analysis of variance.

Latent profile models containing one through six profiles were estimated in Mplus 8.3 using the standardized composite scores for peer trust, peer communication, and peer alienation as continuous indicators. Models were estimated using robust maximum likelihood estimation (MLR). For models with two or more profiles, 1000 initial-stage random starts were generated, the best 250 solutions were retained for final-stage optimization, and 20 initial-stage iterations were used. The bootstrap likelihood ratio test was based on 500 bootstrap draws. Profile-specific indicator means were freely estimated. The variance of each indicator was estimated but constrained to be equal across profiles, whereas all within-profile residual covariances were fixed at zero. The default Mplus convergence criteria were retained. These models were compared to determine the optimal number of profiles. Model selection was based on the following criteria (Collins & Lanza, 2010): (1) Lower values of the Akaike information criterion (AIC), Bayesian information criterion (BIC), and sample-size-adjusted Bayesian information criterion (aBIC) indicated better model fit; (2) Entropy values of 0.80 or higher indicated adequate classification accuracy; (3) significant results from the Vuong–Lo–Mendell–Rubin likelihood ratio test (VLMR), Lo–Mendell–Rubin adjusted likelihood ratio test (LMR), and bootstrap likelihood ratio test (BLRT) indicated that a k-profile model fit the data significantly better than the corresponding k − 1 profile model; and (4) Profile size was also considered, with profiles representing less than 5% of the sample treated as a practical warning (Nylund-Gibson & Choi, 2018). In addition to relying on the aforementioned model fit indices to determine the optimal number of profiles, we also took parsimony and substantive interpretability into account to finalize the most appropriate profile solution (Nylund et al., 2007; Nylund-Gibson & Choi, 2018).

After the optimal number of latent profiles was determined, Welch’s analyses of variance were conducted to examine differences in peer trust, peer communication, and peer alienation across the profiles, followed by Games–Howell post hoc comparisons. Although the individual RCBI items used four ordered response categories, the distal outcomes were 14-item composite mean scores. Consistent with methodological guidance on multi-item composite scores, these scores were treated as approximately continuous (Norman, 2010; Sullivan & Artino, 2013), thereby retaining graded variation in cyberbullying involvement across multiple behaviors. Given the pronounced nonnormality of the outcomes, the BCH models were estimated using MLR. A manual BCH procedure was then used to compare cyberbullying perpetration and cybervictimization across the profiles while accounting for classification error (Asparouhov & Muthén, 2014; Bolck et al., 2004). BCH weights saved from the retained four-profile model were specified as training variables. Age, sex, paternal education, and maternal education were grand-mean centered and entered simultaneously as predictors of both profile membership and each distal outcome, with the two outcomes analyzed in separate models. The relevant Mplus syntax is provided in Supplementary Syntax S1. Following Carter’s (2026) recommendation for applied distal-outcome comparisons in mixture models, the six pairwise comparisons for each distal outcome were treated as a separate family and adjusted using the Benjamini–Hochberg false discovery rate procedure.

Missing data were handled according to the requirements of each analytic stage. For the peer-attachment CFA estimated using MLR, missing item responses were handled using full-information maximum likelihood (FIML). For the RCBI CFAs estimated using WLSMV, missing item responses were handled using the pairwise-present approach implemented in Mplus. The three standardized composite scores used as indicators in the latent profile analysis contained no missing values; therefore, all participants were retained in the LPA. For the BCH distal-outcome analyses, complete-case analysis (listwise deletion) was applied separately to each outcome-specific model. Given the very small proportions of excluded cases (0.1% and 0.3%, respectively), this approach resulted in minimal loss of sample size.

Among the 1851 participants retained at follow-up, Little’s MCAR test was conducted on the five composite study variables: Time 1 peer trust, peer communication, and peer alienation, and Time 2 cyberbullying perpetration and cybervictimization. The test was nonsignificant, χ^2^(7) = 5.45, *p* = 0.606, indicating that the remaining variable-level missingness across these variables was consistent with the MCAR assumption. The study and its analytic plan were not preregistered.

## 3. Results

### 3.1. Common Method Bias Test

Because all study variables were assessed using self-report measures, common method bias remained a potential concern. The study addressed this concern through both a procedural remedy and a statistical diagnostic. Procedurally, a six-month temporal separation was introduced between the assessment of peer attachment and the assessment of cyberbullying outcomes. This separation may have reduced both participants’ ability to recall and deliberately align their responses across measures and the influence of shared transient mood and contextual effects (Podsakoff et al., 2012). Statistically, Harman’s single-factor test was used only as a limited diagnostic of potential common method bias. The analysis yielded 12 factors with eigenvalues greater than 1, and the first factor accounted for 23.44% of the total variance, which was below the commonly used 40% benchmark (D. Tang & Wen, 2020). This result indicates only that the data did not exhibit an obvious dominant single-factor structure. Taken together, these procedural and statistical considerations reduce, but do not eliminate, concerns regarding common-method variance.

### 3.2. Descriptive Statistics

Table 1 presents the descriptive statistics, distributional characteristics, and correlations among the main study variables. Peer trust and peer communication were negatively correlated with both cyberbullying perpetration and cybervictimization, whereas peer alienation was positively correlated with both outcomes. Cyberbullying perpetration and cybervictimization were also positively correlated. The peer attachment dimensions showed relatively limited departures from normality. In contrast, cyberbullying perpetration and cybervictimization exhibited pronounced positive skewness and kurtosis, indicating substantial concentration toward the lower end of their respective distributions.

Given the relatively strong correlation between cyberbullying perpetration and cybervictimization (*r* = 0.54), a cross-tabulation was conducted to examine the overlap between the two forms of involvement. Following the classification approach used in previous research (Song & Wang, 2026), participants with complete data on both subscales were classified into four mutually exclusive groups: non-involved, victim-only, perpetrator-only, and bully–victim. For each subscale, a total score of 14 indicated no involvement, whereas a score greater than 14 indicated any involvement. Of the 1851 participants, 1810 (97.8%) had complete data on both subscales and were included in the analysis. As shown in Table 2, 43.3% were classified as non-involved, 20.2% as victim-only, 10.4% as perpetrator-only, and 26.2% as bully–victims. Cyberbullying perpetration was significantly associated with cybervictimization, χ^2^(1) = 267.55, *p* < 0.001, φ = 0.38. Specifically, 71.6% of participants who reported perpetration also reported victimization, whereas 56.5% of those who reported victimization also reported perpetration, indicating substantial but incomplete overlap between the two forms of cyberbullying involvement.

### 3.3. Latent Profile Analysis

Latent profile analysis was conducted using the standardized composite scores for peer trust, peer communication, and peer alienation as continuous indicators. Solutions with one to six profiles were estimated and compared. As shown in Table 3, the AIC, BIC, and sample-size-adjusted BIC (aBIC) decreased as additional profiles were extracted. The four-profile solution yielded an entropy value of 0.889, indicating adequate classification accuracy. Moreover, the VLMR, adjusted LMR, and BLRT all indicated that the four-profile solution provided a significantly better fit than the three-profile solution (all *ps* < 0.001). The information criteria continued to decrease for the five- and six-profile solutions, and their entropy values increased slightly. The VLMR, adjusted LMR, and BLRT also remained significant for both the five- and six-profile solutions (all *ps* < 0.001). Nevertheless, the incremental improvement in fit became substantially smaller after four profiles. For example, the BIC decreased by 788.17 points from the three-profile to the four-profile solution, compared with decreases of 196.10 points from the four-profile to the five-profile solution and 128.81 points from the five-profile to the six-profile solution. This pattern suggested diminishing improvements in relative fit beyond four profiles. Profile size and parsimony provided additional grounds for retaining the four-profile solution. Based on most likely profile membership, the smallest profile in the four-profile solution contained 159 participants (8.59%). In contrast, the smallest profiles contained only 39 participants (2.11%) in the five-profile solution and 18 participants (0.97%) in the six-profile solution. The very small absolute and relative sizes of these profiles raised concerns regarding the precision and stability of their parameter estimates and their suitability for subsequent distal-outcome comparisons. The 5% benchmark was not treated as a rigid exclusion criterion; rather, it was used as a practical warning and considered jointly with absolute profile size, relative model fit, classification accuracy, parsimony, and theoretical interpretability (Nylund-Gibson & Choi, 2018; Sorgente et al., 2025).

**Figure 1:**
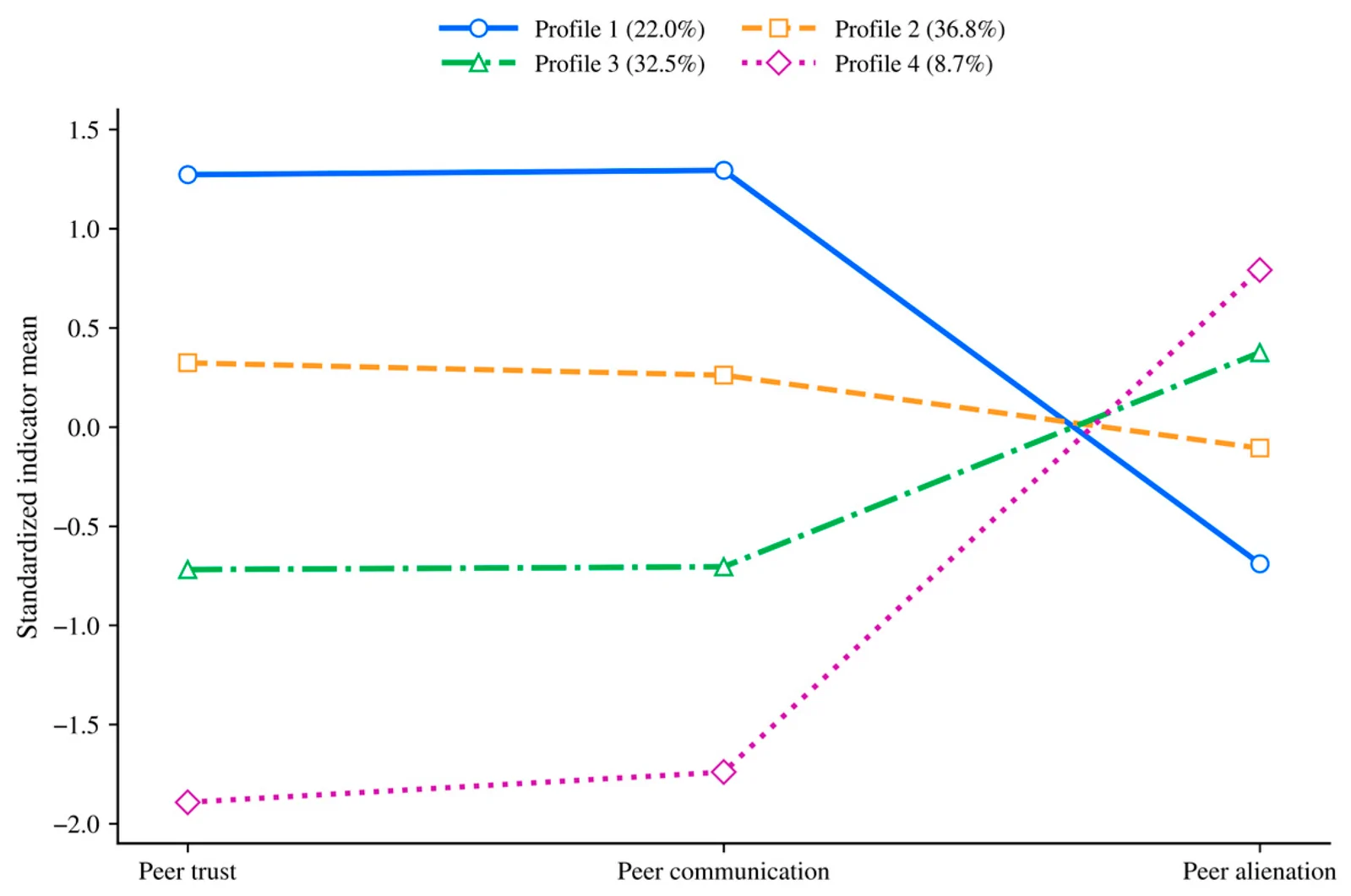
Four-profile solution of peer attachment among adolescents.

Accordingly, the four-profile model was retained primarily because it offered the most useful balance of parsimony, profile size, and interpretability rather than because statistical fit indices unequivocally identified four profiles. The characteristics of the four latent profiles are illustrated in Figure 1, in which the percentages represent model-estimated profile proportions based on posterior membership probabilities. In addition, the average posterior probabilities of membership in the assigned profiles ranged from 93.4% to 94.8%, indicating good classification accuracy for the four-profile solution. The retained four-profile model converged normally, and the best log-likelihood value was replicated in all 250 final-stage optimizations (log-likelihood = −6236.837), supporting the stability of the solution and reducing concern that the model had converged on a local maximum.

The four profiles were differentiated primarily by their overall level of peer-attachment security rather than by sharply contrasting configurations. Across the four profiles, peer trust and peer communication decreased progressively, whereas peer alienation increased progressively. Profile 1 was characterized by the highest levels of peer trust and peer communication and the lowest level of peer alienation. Its scores on peer trust and peer communication were above the corresponding overall sample means (*M* = 3.66 and *M* = 3.45, respectively), whereas its score on peer alienation was below the overall sample mean (*M* = 2.16), suggesting the most secure configuration of peer attachment. In contrast, Profiles 3 and 4 both showed peer trust and peer communication scores below the overall sample means and peer alienation scores above the overall sample mean, suggesting relatively less secure configurations of peer attachment. Profile 3 showed a relatively low attachment pattern, and Profile 4 showed the least favorable pattern. Profile 2 occupied an intermediate position across all three dimensions, with its scores consistently falling between those of Profile 1 and Profiles 3 and 4. Taken together, these relative configurations provided the basis for labeling the four profiles. The specific characteristics and corresponding labels of each profile are described below.

Profile 1 comprised 408 adolescents (22.04%). Adolescents in this profile had the highest scores on peer trust (*M* = 4.79) and peer communication (*M* = 4.60) and the lowest score on peer alienation (*M* = 1.63). Accordingly, this profile was labeled the secure profile. Profile 2 comprised 678 adolescents (36.63%) and represented the largest profile in the sample. Adolescents in this profile showed relatively high scores on peer trust (*M* = 3.95) and peer communication (*M* = 3.69), with both scores lower than those of the secure profile but higher than those of the low attachment and alienated profiles. Their score on peer alienation (*M* = 2.09) was higher than that of the secure profile but lower than those of the low attachment and alienated profiles. Because this configuration consistently occupied an intermediate position between the most secure profile and the two relatively less secure profiles, it was labeled the intermediate profile. Profile 3 comprised 606 adolescents (32.74%). Adolescents in this profile reported relatively low levels of peer trust (*M* = 3.02) and peer communication (*M* = 2.82), both of which were below the corresponding overall sample means, together with a comparatively elevated level of peer alienation (*M* = 2.44). Compared with the secure and intermediate profiles, this profile was characterized by fewer positive peer-attachment resources and stronger feelings of alienation; however, these characteristics were less extreme than those observed in the alienated profile. This pattern therefore reflected a generally low, but not the lowest, level of peer-attachment quality and was labeled the low attachment profile. Although some trust and communication remained, these positive relational experiences coexisted with elevated feelings of alienation, suggesting a degree of relational tension. Profile 4 comprised 159 adolescents (8.59%). Adolescents in this profile had the lowest scores on peer trust (*M* = 1.97) and peer communication (*M* = 1.89) and the highest score on peer alienation (*M* = 2.75), reflecting a configuration of low trust and communication and high alienation. Accordingly, this profile was labeled the alienated profile. To further examine differences among the four latent profiles across the dimensions of peer attachment, post hoc comparisons were conducted. As shown in Table 4, significant differences were found among the four profiles across all dimensions of peer attachment, supporting clear differentiation among the four profiles.

**Figure 2:**
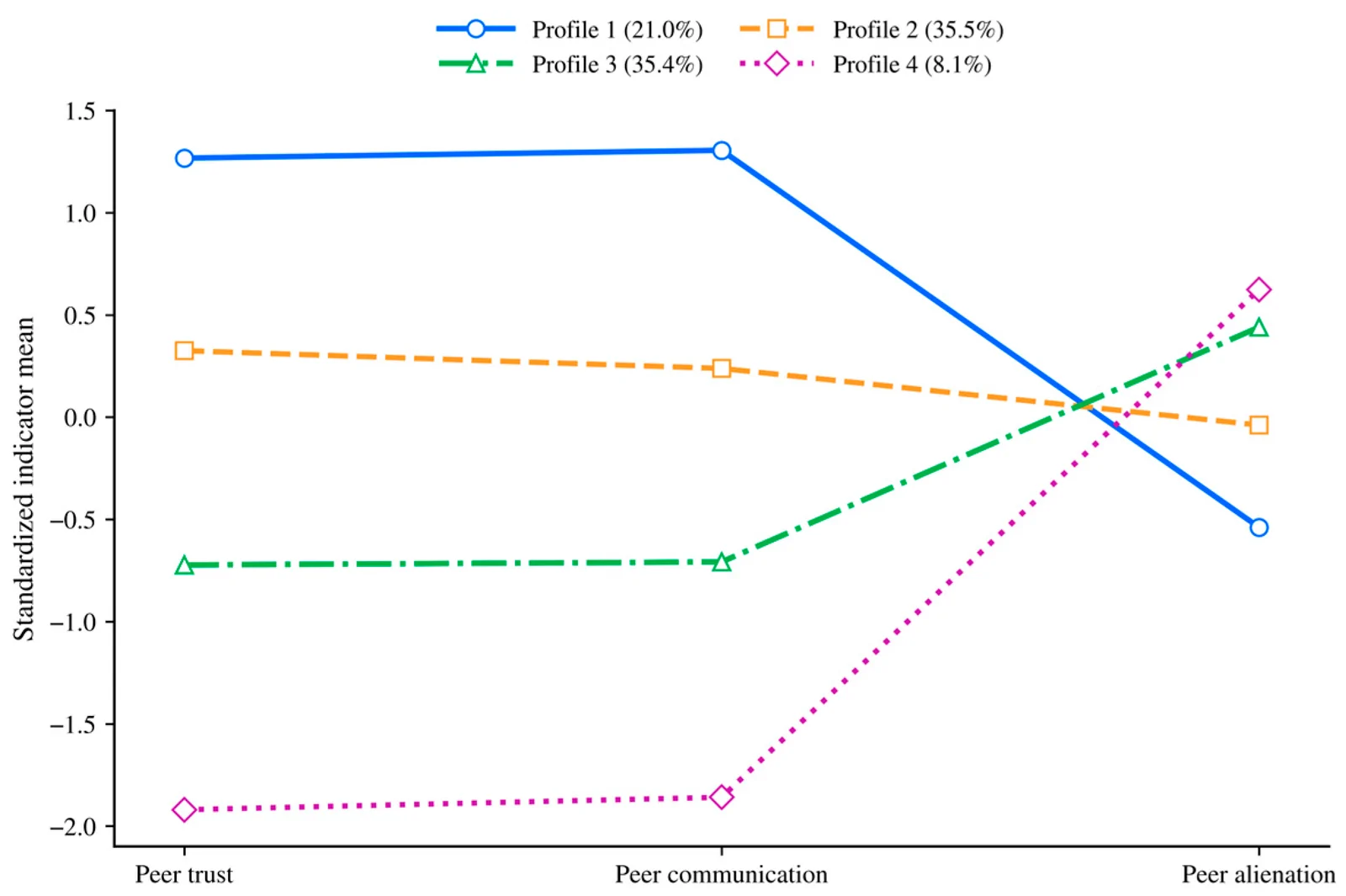
Four-profile solution of peer attachment among boys.

**Figure 3:**
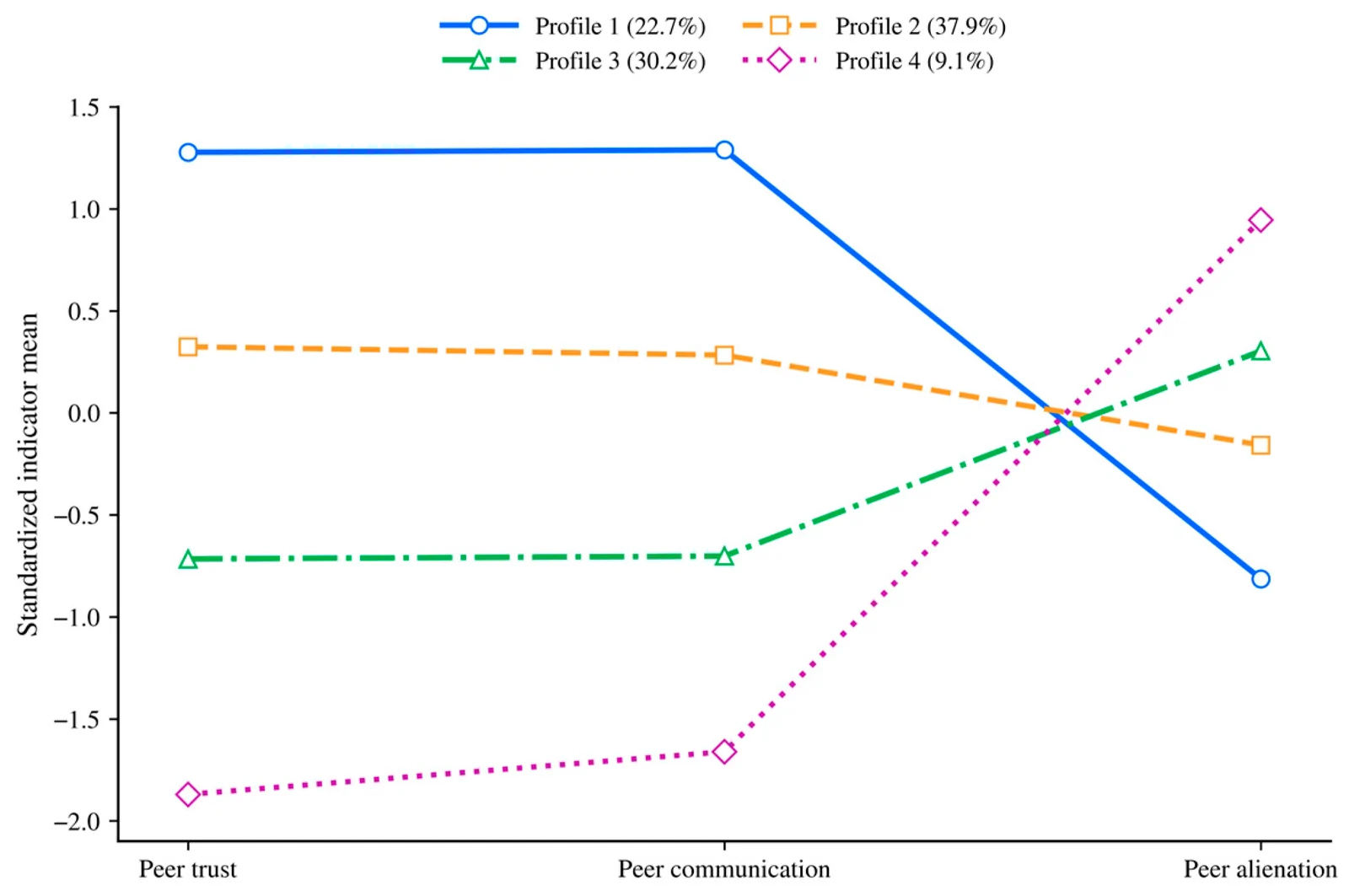
Four-profile solution of peer attachment among girls.

To explore whether similar latent profile structures of peer attachment would emerge independently among boys and girls, separate latent profile analyses were conducted for each gender group, following previous research (Irvin-Vitela et al., 2026). As shown in Table 5 and Figure 2 and Figure 3, the same model-selection criteria used for the full sample were applied to the gender-specific analyses. The four-profile solutions showed adequate classification accuracy, with entropy values of 0.873 for boys and 0.904 for girls, and the VLMR, adjusted LMR, and BLRT were all significant (all *ps* < 0.001). Their smallest profiles represented 7.69% of boys and 8.95% of girls. Although the information criteria continued to decrease for the five- and six-profile solutions, these solutions produced very small profiles, representing only 1.42–1.89% of boys and 0.60–1.99% of girls. Considering relative model fit, classification accuracy, profile size, parsimony, and substantive interpretability, the four-profile solution was therefore retained for both gender groups. The average posterior probabilities of membership in the assigned profiles ranged from 92.0% to 94.8% for boys and from 94.3% to 95.7% for girls, indicating good classification accuracy in both groups. Overall, similar four-profile solutions emerged independently among boys and girls and were broadly comparable to the full-sample solution.

### 3.4. Differences Across Peer Attachment Profiles in Subsequent Cyberbullying Perpetration and Cybervictimization

To examine whether Time 1 peer attachment profiles were prospectively associated with cyberbullying perpetration and cybervictimization assessed six months later, the BCH method was used to compare these continuous distal outcomes across profiles while accounting for classification uncertainty. The available demographic variables included age, grade, sex, paternal education, and maternal education. Because age and grade were highly correlated and represented substantially overlapping information about adolescents’ developmental and educational stages, they were not entered simultaneously to avoid redundancy and potential multicollinearity. Age was retained because it provided a continuous and more fine-grained indicator of developmental stage. Accordingly, age, sex, paternal education, and maternal education were included as covariates in the distal-outcome analyses. As shown in Table 6, after accounting for these covariates, the omnibus Wald tests indicated significant differences across the four profiles in cyberbullying perpetration (Wald χ^2^(3) = 36.360, *p* < 0.001) and cybervictimization (Wald χ^2^(3) = 58.276, *p* < 0.001).

Further pairwise comparisons for cyberbullying perpetration and cybervictimization are presented in Table 7. After applying the Benjamini–Hochberg false discovery rate correction to the six comparisons conducted for each outcome, adolescents in the secure profile reported significantly lower levels of both cyberbullying perpetration and cybervictimization than those in the intermediate, low attachment, and alienated profiles. Adolescents in the intermediate profile also reported significantly lower levels of both outcomes than those in the low attachment and alienated profiles. However, the low attachment and alienated profiles did not differ significantly in cyberbullying perpetration (adjusted *p* = 0.658, *d* = −0.046, 95% CI [−0.220, 0.128]) or cybervictimization (adjusted *p* = 0.089, *d* = −0.168, 95% CI [−0.342, 0.006]). Across the statistically significant comparisons, the absolute effect sizes ranged from 0.172 to 0.377 for cyberbullying perpetration and from 0.198 to 0.582 for cybervictimization, indicating predominantly small effects for perpetration and small to moderate effects for victimization.

## 4. Discussion

The present study adopted a person-centered approach to examine latent profiles of peer attachment among Chinese adolescents and their prospective associations with cyberbullying perpetration and cybervictimization. Empirically, four peer attachment profiles were identified: secure, intermediate, low attachment, and alienated. Similar four-profile solutions emerged independently in the separate analyses of boys and girls, providing exploratory evidence of descriptive similarity across gender groups. In addition, adolescents with different peer attachment profiles differed significantly in their levels of cyberbullying perpetration and cybervictimization six months later. Specifically, adolescents in the secure profile showed the lowest levels of both outcomes, followed by those in the intermediate profile, whereas adolescents in the low attachment and alienated profiles showed higher levels, with no significant differences between the latter two profiles.

### 4.1. Latent Profiles of Adolescent Peer Attachment

The four peer attachment profiles identified in the present study highlight substantial heterogeneity in peer attachment among Chinese adolescents, consistent with previous research (X. Q. Li et al., 2009). In essence, the secure profile was characterized by the predominance of positive relational features, with high trust and communication and low alienation. The intermediate profile occupied a middle position between the more and less secure configurations. The low attachment profile showed the least differentiation among trust, communication, and alienation, reflecting a relatively balanced configuration in which positive and negative attachment characteristics were similarly represented. In contrast, the alienated profile was characterized by low trust and communication and high alienation. The four profiles differed significantly across all dimensions of peer attachment, with differences being more pronounced for the positive dimensions of peer trust and peer communication than for the negative dimension of peer alienation. This pattern may indicate that the positive dimensions of peer attachment play a particularly important role in differentiating adolescents’ peer attachment profiles. In addition, similar four-profile solutions emerged independently in the separate analyses of boys and girls, suggesting that the overall pattern was not confined to either gender group. Together, these findings provide a more differentiated framework for understanding peer attachment among Chinese adolescents.

The intermediate profile represented the largest proportion of adolescents in the present study. This pattern differs from findings in research on parent–child attachment in infancy and partner attachment in adulthood, in which secure attachment is typically the predominant pattern (Deneault et al., 2023; X. Li et al., 2023). One possible interpretation is that the predominance of the intermediate profile reflects the transitional nature of adolescent peer attachment, which may still be undergoing integration and reorganization, rather than indicating an atypical attachment pattern simply because it is less secure than the secure profile. Attachment stability varies across developmental periods. Compared with parent–child attachment established early in life and partner attachment in adulthood, attachment representations during adolescence may be more susceptible to developmental transitions and ongoing relational experiences (Dinero et al., 2022; Fraley & Roisman, 2019; Opie et al., 2021). Longitudinal evidence also suggests that attachment is less stable during adolescence than during adulthood and may be more likely to change with relational experiences under conditions such as family conflict, parental separation, and minority status (Jones et al., 2018; Khan et al., 2020). In addition, factors such as parent attachment, teacher–student relationships, and internalizing problems may also be associated with adolescents’ representations of peer attachment (Delgado et al., 2022; Sun et al., 2025; Therriault et al., 2024).

From this developmental perspective, adolescents in the intermediate profile may already experience meaningful levels of trust and communication in their peer relationships while showing a less consistently secure configuration than adolescents in the secure profile. Their levels of trust and communication were higher, and their level of alienation was lower, than those of adolescents in the low attachment and alienated profiles. Thus, the relatively high prevalence of the intermediate profile may reflect the dynamic and still-developing nature of peer attachment during adolescence rather than an atypical form of attachment.

### 4.2. Prospective Associations of Peer Attachment Profiles with Cyberbullying Perpetration and Cybervictimization

The present study found that peer attachment profiles were significantly associated with adolescents’ levels of cyberbullying perpetration and cybervictimization six months later. Both outcomes showed a similar pattern: adolescents in the secure profile had the lowest levels, those in the intermediate profile showed higher levels, and those in the low attachment and alienated profiles showed the highest levels, with no significant differences between the latter two profiles. This pattern is consistent with previous research linking stronger peer attachment and peer support to lower involvement in cyber aggression and cybervictimization (Arató et al., 2022; Wright et al., 2015). These findings suggest that more favorable configurations of peer attachment may constitute an important relational resource associated with lower subsequent levels of both forms of cyberbullying involvement.

Although the processes underlying these associations were not directly assessed in the present study, several theoretically plausible explanations may help contextualize the observed pattern. Secure peer attachment is characterized by trust, open communication, and low alienation, which may be associated with stable interpersonal connections, greater emotional support, and more positive interpersonal representations. Attachment theory proposes that secure attachment is associated with more positive internal working models, which are related to how individuals process social information and approach subsequent interpersonal interactions (Dykas & Cassidy, 2011). From this perspective, adolescents in the secure profile may be more likely to interpret others’ online behavior as less threatening and to respond constructively. Such tendencies could be associated with fewer misunderstandings and hostile attributions, as well as less intense negative emotional reactions during interpersonal interactions. Previous research has also shown that fewer difficulties in emotion regulation and better socioemotional adjustment are associated with lower levels of cyberbullying perpetration (Arató et al., 2022; Moreta-Herrera et al., 2025). Accordingly, the lower level of cyberbullying perpetration observed in the secure profile may be related to fewer negative response tendencies in online interpersonal interactions and more favorable socioemotional adjustment.

With respect to cybervictimization, previous research has identified social support as an important relational resource associated with lower levels of cybervictimization among adolescents (Kowalski et al., 2014). One possible explanation is that secure peer attachment may be associated with more stable and supportive peer relationships, which could offer greater emotional support and a stronger sense of social connectedness (Schoeps et al., 2020). These relational resources may be relevant to cybervictimization in two ways. First, supportive peer relationships may be associated with lower interpersonal vulnerability in negative online interactions by providing a stronger social support network. Second, when negative online experiences occur, such relationships may provide adolescents with greater opportunities to seek support and cope effectively with these experiences. These relational resources therefore offer one possible explanation for why adolescents in the secure profile reported lower levels of cybervictimization six months later.

Adolescents in the low attachment and alienated profiles reported significantly higher levels of both cyberbullying perpetration and cybervictimization than those in the secure and intermediate profiles, whereas no significant differences emerged between the low attachment and alienated profiles. The low attachment profile was characterized by below-average peer trust and communication and above-average peer alienation, although these difficulties were less pronounced than those observed in the alienated profile. This profile therefore reflected an overall unfavorable pattern across all three dimensions of peer attachment. One possible interpretation is that adolescents in this profile may have less confidence in their peers’ availability and responsiveness and fewer opportunities to express concerns, seek support, clarify misunderstandings, and resolve interpersonal conflicts. These difficulties may be particularly salient in online contexts, where nonverbal cues are limited and ambiguous messages may be more readily interpreted as hostile (Demircioğlu & Göncü-Köse, 2023). Accordingly, lower trust and communication may be associated with misunderstandings and reactive responses, which may correspond to more frequent cyberbullying perpetration. Heightened alienation and a reduced tendency to seek support may also be associated with higher levels of cybervictimization.

Adolescents in the alienated profile were characterized by low peer trust and communication and high peer alienation. Longitudinal evidence has similarly shown that peer alienation is positively associated with subsequent cyberbullying and cybervictimization (Charalampous et al., 2018), supporting the relevance of alienated peer relationships to later cyberbullying involvement. From an attachment perspective, more alienated relational patterns may be accompanied by greater emotional suppression, reduced emotional involvement, and greater avoidance of close interpersonal interactions (Dagan et al., 2021; Mikulincer & Shaver, 2016). Although such strategies may be associated with less direct interpersonal conflict offline, they may also coincide with fewer opportunities for positive communication, help-seeking, and conflict repair in online interactions. When confronted with aggression, adolescents who rely on emotional disengagement or passive avoidance may be less likely to seek support or actively address interpersonal difficulties, which may be associated with continued experiences of victimization. Their detached or indirect responses may also be associated with less effective conflict resolution and continued involvement in negative online interactions. Thus, the elevated cyberbullying involvement observed in the two profiles may reflect different relational difficulties—broadly diminished trust and communication in the low attachment profile and more pronounced relational withdrawal in the alienated profile—although these possibilities require direct empirical examination.

Adolescents in the intermediate profile showed levels of cyberbullying perpetration and cybervictimization that fell between those of the secure profile and the two insecure profiles. This pattern suggests that their comparatively more favorable peer relationship resources may be associated with lower levels of cyberbullying involvement than those observed in the less secure profiles, although these resources may be less stable or sufficient than those available to adolescents in the secure profile.

Although the omnibus differences were statistically significant, the pairwise effect sizes were predominantly small for cyberbullying perpetration and small to moderate for cybervictimization. These profile differences should be interpreted in light of the large sample size and the pronounced floor effects in both outcomes. Nevertheless, the cross-classification results showed that 56.7% of participants reported at least one form of cyberbullying involvement, indicating that such involvement was not negligible despite the pronounced floor effects in both outcome scores.

### 4.3. Implications

At the theoretical level, the present study extends the application of attachment theory to adolescents’ online behavior. Attachment theory emphasizes that representations of the self and others formed within important relationships shape how individuals interpret interpersonal cues, regulate emotions, and respond to interpersonal conflict (Bowlby, 1988). During adolescence, peers increasingly become important sources of emotional support, social recognition, and relational security. From this perspective, adolescents’ online interpersonal behavior may be related to relational experiences within their offline peer relationships rather than being entirely independent of them. By adopting a person-centered approach, the present study identified four empirically derived peer attachment profiles that differed primarily in their overall level of attachment quality. Similar patterns of prospective associations were observed between the Time 1 peer attachment profiles and both cyberbullying perpetration and cybervictimization assessed six months later. These findings not only deepen understanding of heterogeneity in adolescent peer attachment but also provide a person-centered perspective on the relational factors shared by adolescents involved in cyberbullying perpetration and cybervictimization.

At the practical level, the findings highlight peer attachment as a potentially relevant relational context for future cyberbullying prevention research. The higher subsequent levels of cyberbullying perpetration and cybervictimization observed in the low attachment and alienated profiles suggest that assessing peer trust, communication, and alienation may complement the assessment of adolescents’ online behavior. Peer attachment may therefore be considered a potential target for future intervention studies. However, because the present study did not evaluate an intervention or assess baseline cyberbullying involvement, whether strengthening peer attachment can reduce subsequent cyberbullying remains to be tested. In addition, the similar patterns observed for perpetration and victimization suggest that both forms of involvement may be considered within a coordinated assessment and prevention framework.

### 4.4. Limitations and Future Directions

Several limitations should be considered when interpreting these findings.

First, cyberbullying perpetration and cybervictimization were assessed only at T2 and were not measured at T1. Consequently, the analyses could not control for baseline levels or determine whether peer attachment profiles were associated with changes in these outcomes. Although the temporal ordering of the measurements supports a prospective association, it does not establish causality or rule out the possibility that cyberbullying experiences occurring before T1 influenced adolescents’ peer attachment. Future studies should assess both peer attachment and cyberbullying involvement at multiple time points to examine baseline-adjusted changes and potential reciprocal associations.

Second, both cyberbullying outcomes exhibited pronounced floor effects, with substantial proportions of participants scoring at the theoretical minimum. This concentration restricted variability at the lower end of the distributions and may have reduced sensitivity to differences between profiles. The observed differences should therefore be interpreted as relative differences within generally low-frequency distributions rather than as large absolute differences in cyberbullying behavior. In addition, all variables were assessed using adolescent self-reports and may have been affected by recall or social desirability bias. Future research could incorporate reports from peers, teachers, or parents and recruit samples with greater variation in cyberbullying involvement.

Third, the Chinese sociocultural context may also be relevant to the interpretation of these findings. Cultural norms can shape peer evaluations and interactions, while group belonging and supportive peer relationships represent important sources of security and socioemotional adjustment, particularly in relatively group-oriented contexts (X. Chen, 2012). Moreover, Chinese adolescents may experience substantial academic and peer-related pressures (X. Zhou et al., 2023), while platforms such as WeChat, QQ, Weibo, and short-video applications provide important settings in which social interactions and cyberbullying involvement may occur (L. Tang et al., 2021). These contextual characteristics may increase the relevance of peer trust, communication, and alienation to adolescents’ online experiences. However, the sample consisted exclusively of adolescents from Shandong Province, China, and cultural values, academic pressure, and platform-specific social media use were not directly assessed. Caution is therefore warranted when generalizing the findings to adolescents from other regions or cultural contexts.

Fourth, the present study examined prospective associations between peer attachment profiles and cyberbullying perpetration and cybervictimization assessed six months later but did not assess the processes underlying these associations. Future research could incorporate potential mediating and moderating variables to clarify the pathways through which peer attachment profiles are associated with cyberbullying perpetration and cybervictimization, as well as the conditions under which these associations may vary.

Finally, the relatively short follow-up period and the assessment of peer attachment at only one time point limited our ability to examine its longer-term developmental dynamics. Future studies should employ longer follow-up periods and repeated assessments of peer attachment to investigate profile stability and transitions over time.

## 5. Conclusions

Overall, using a person-centered approach, the present study identified four latent profiles of peer attachment among Chinese adolescents: the secure, intermediate, low attachment, and alienated profiles, highlighting meaningful heterogeneity in adolescent peer attachment. Similar four-profile solutions also emerged in separate analyses of boys and girls. The profiles primarily reflected graded differences in overall peer-attachment quality. Time 1 peer attachment profiles were prospectively associated with cyberbullying perpetration and cybervictimization assessed six months later. Adolescents in the secure profile showed the lowest levels of both outcomes, followed by those in the intermediate profile, whereas adolescents in the low attachment and alienated profiles showed the highest levels, with no significant differences between the latter two profiles. These findings describe temporal associations rather than changes or causal effects. They may inform future research on profile-informed assessment and prevention.

## Figures and Tables

**Table 1:** Descriptive statistics and correlations for the main variables.

Variable	*M*	*SD*	*Skewness*	*Kurtosis*	1	2	3	4	5
1. Peer trust	3.66	0.89	−0.38	−0.38	—				
2. Peer communication	3.45	0.88	−0.18	−0.47	0.889 ***	—			
3. Peer alienation	2.16	0.75	0.61	0.21	−0.463 ***	−0.391 ***	—		
4. Cyberbullying perpetration	1.09	0.22	4.27	21.80	−0.151 ***	−0.125 ***	0.150 ***	—	
5. Cybervictimization	1.16	0.33	3.34	13.70	−0.186 ***	−0.166 ***	0.213 ***	0.545 ***	—

Note. *** *p* < 0.001.

**Table 2:** Cross-Classification of Cyberbullying Perpetration and Cybervictimization.

Cyberbullying Perpetration	No Cybervictimization	Cybervictimization	Total
No perpetration	783 (43.3%) Non-involved	365 (20.2%) Victim-only	1148 (63.4%)
Perpetration	188 (10.4%) Perpetrator-only	474 (26.2%) Bully–victim	662 (36.6%)
Total	971 (53.6%)	839 (46.4%)	1810 (100.0%)

Note. Values are presented as *n* (% of the valid analytic sample). Percentages were calculated among the 1810 participants with complete data on both cyberbullying subscales. Forty-one participants (2.2% of the original sample) were excluded because their involvement status could not be determined due to missing data. A subscale score of 14 indicated no involvement, whereas a score greater than 14 indicated any involvement.

**Table 3:** Fit indices of latent profile analysis for peer attachment.

Profile	AIC	BIC	aBIC	Entropy	VLMR *p*	LMR *p*	BLRT *p*	Smallest Profile Size (*n*, %)
1	15,767.73	15,800.87	15,781.81	—	—	—	—	1851 (100.00%)
2	13,971.29	14,026.52	13,994.75	0.820	<0.001	<0.001	<0.001	843 (45.54%)
3	13,319.95	13,397.27	13,352.80	0.800	<0.050	<0.050	<0.001	460 (24.85%)
**4**	**12,509.67**	**12,609.10**	**12,551.91**	**0.889**	**<0.001**	**<0.001**	**<0.001**	**159 (8.59%)**
5	12,291.49	12,413.00	12,343.11	0.897	<0.001	<0.001	<0.001	39 (2.11%)
6	12,140.58	12,284.19	12,201.58	0.905	<0.001	<0.001	<0.001	18 (0.97%)

Note. Bold values indicate the fit indices used to determine the final four-profile solution. AIC = Akaike Information Criterion; BIC = Bayesian Information Criterion; aBIC = sample-size adjusted Bayesian Information Criterion; VLMR *p* = Vuong-Lo-Mendell-Rubin likelihood ratio test *p*-value; LMR *p* = Lo-Mendell-Rubin adjusted likelihood ratio test *p*-value; BLRT *p* = Bootstrapped Likelihood Ratio Test *p*-value; Smallest Profile Size = size and percentage of the smallest extracted class based on most likely class membership.

**Table 4:** Descriptive statistics and comparisons across peer attachment profiles.

	Peer Trust	Peer Communication	Peer Alienation
	*M* (*SD*)	*M* (*SD*)	*M* (*SD*)
SEC (*n* = 408)	4.79 (0.22)	4.60 (0.34)	1.63 (0.70)
INT (*n* = 678)	3.95 (0.28)	3.69 (0.35)	2.09 (0.66)
LA (*n* = 606)	3.02 (0.33)	2.82 (0.38)	2.44 (0.60)
ALI (*n* = 159)	1.97 (0.41)	1.89 (0.42)	2.75 (0.84)
Welch’s *F*	4854.19 ***	2849.56 ***	148.50 ***
η^2^_p_	0.887	0.830	0.211
Games–Howell post hoc	SEC > INT > LA > ALI	SEC > INT > LA > ALI	SEC < INT < LA< ALI

Note. *** *p* < 0.001. SEC = Secure profile, INT = Intermediate profile, LA = Low attachment profile, ALI = Alienated profile. All pairwise differences in the Games–Howell tests were statistically significant at *p* < 0.001.

**Table 5:** Fit indices for latent profile analyses of peer attachment among boys and girls.

Profile	AIC	BIC	aBIC	Entropy	VLMR *p*	LMR *p*	BLRT *p*	Smallest Profile Size (*n*, %)
Boys								
1	7298.57	7327.00	7307.95	—	—	—	—	845 (100.00%)
2	6577.12	6624.52	6592.76	0.801	<0.001	<0.001	<0.001	413 (48.88%)
3	6299.53	6365.88	6321.42	0.823	<0.050	<0.050	<0.001	94 (11.12%)
**4**	**5989.45**	**6074.76**	**6017.60**	**0.873**	**<0.001**	**<0.001**	**<0.001**	**65 (7.69%)**
5	5899.84	6004.10	5934.24	0.889	<0.050	<0.050	<0.001	16 (1.89%)
6	5825.72	5948.94	5866.37	0.895	<0.001	<0.001	<0.001	12 (1.42%)
Girls								
1	8459.88	8489.37	8470.31	—	—	—	—	1006 (100.00%)
2	7375.54	7424.68	7392.92	0.837	<0.001	<0.001	<0.001	431 (42.84%)
3	6961.61	7030.40	6985.94	0.828	<0.010	<0.010	<0.001	247 (24.55%)
**4**	**6482.03**	**6570.48**	**6513.31**	**0.904**	**<0.001**	**<0.001**	**<0.001**	**90 (8.95%)**
5	6364.48	6472.58	6402.70	0.908	<0.010	<0.010	<0.001	20 (1.99%)
6	6301.17	6428.93	6346.35	0.917	<0.001	<0.001	<0.001	6 (0.60%)

Note. Boys: *n* = 845; girls: *n* = 1006. AIC = Akaike information criterion; BIC = Bayesian information criterion; aBIC = sample-size-adjusted Bayesian information criterion; LMR = Lo–Mendell–Rubin adjusted likelihood ratio test; BLRT = bootstrap likelihood ratio test. Bold values indicate the fit indices used to determine the final four-profile solution.

**Table 6:** Differences in cyberbullying perpetration and cybervictimization across peer attachment profiles.

Variables	SEC	INT	LA	ALI	Wald χ^2^
	*M* (*SE*)	*M* (*SE*)	*M* (*SE*)	*M* (*SE*)	
Cyberbullying perpetration	1.051 (0.008)	1.080 (0.008)	1.128 (0.012)	1.141 (0.025)	36.360 ***
Cybervictimization	1.090 (0.011)	1.139 (0.012)	1.212 (0.017)	1.281 (0.035)	58.276 ***

Notes. *** *p* < 0.001. SEC = Secure profile, INT = Intermediate profile, LA = Low attachment profile, ALI = Alienated profile.

**Table 7:** Pairwise comparisons of cyberbullying perpetration and cybervictimization across peer attachment profiles.

Comparison	Mean Difference	Unadjusted *p*	BH/FDR-Adjusted *p*	Cohen’s *d*	95% CI for *d*
cyberbullying perpetration					
SEC vs. LA	−0.077	<0.001	<0.001	−0.351	[−0.478, −0.225]
SEC vs. ALI	−0.090	<0.001	0.002	−0.377	[−0.561, −0.193]
INT vs. SEC	0.029	0.015	0.022	0.172	[0.049, 0.295]
INT vs. LA	−0.048	<0.001	0.002	−0.209	[−0.320, −0.099]
INT vs. ALI	−0.061	0.022	0.027	−0.246	[−0.418, −0.074]
LA vs. ALI	−0.013	0.658	0.658	−0.046	[−0.220, 0.128]
cybervictimization					
SEC vs. LA	−0.122	<0.001	<0.001	−0.391	[−0.518, −0.264]
SEC vs. ALI	−0.190	<0.001	<0.001	−0.582	[−0.768, −0.397]
INT vs. SEC	0.048	0.003	0.004	0.198	[0.075, 0.321]
INT vs. LA	−0.074	<0.001	0.001	−0.217	[−0.327, −0.107]
INT vs. ALI	−0.142	<0.001	<0.001	−0.401	[−0.574, −0.228]
LA vs. ALI	−0.068	0.089	0.089	−0.168	[−0.342, 0.006]

Notes. SEC = Secure profile, INT = Intermediate profile, LA = Low attachment profile, ALI = Alienated profile. Mean differences equal the covariate-adjusted mean of the first profile minus that of the second profile. Means were adjusted for age, sex, paternal education, and maternal education. Unadjusted *p* values were obtained from pairwise Wald chi-square tests of the profile-specific intercepts. BH/FDR-adjusted *p* values were calculated across the six comparisons using the Benjamini–Hochberg procedure. Cohen’s *d* is the standardized mean difference based on profile-specific within-profile variances. BH/FDR = Benjamini–Hochberg false discovery rate; CI = confidence interval. Values of *p* below 0.001 are reported as <0.001.

## Data Availability

The data and materials supporting the findings of this study are available from the corresponding author upon reasonable request.

## Supplementary Materials

The following supporting information can be downloaded at: https://www.mdpi.com/article/10.3390/bs16101852/s1, Supplementary Syntax S1. Mplus Syntax for the Covariate-Adjusted Manual BCH Analysis of Cyberbullying Perpetration and Cybervictimization.

## Abbreviations

The following abbreviations are used in this manuscript:

LPA	latent profile analysis
AIC	Akaike information criterion
BIC	Bayesian information criterion
aBIC	Sample-size-adjusted Bayesian information criterion
VLMR	Vuong–Lo–Mendell–Rubin likelihood ratio test
LMR	Lo–Mendell–Rubin likelihood ratio test
BLRT	bootstrap likelihood ratio test
BCH	Bolck–Croon–Hagenaars
SEC	Secure profile
INT	Intermediate profile
LA	Low attachment profile
ALI	Alienated profile
