# Supplementary material for: Latent Profiles of Peer Attachment and Their Longitudinal Associations with Cyberbullying Perpetration and Cybervictimization Among Chinese Adolescents
Source: Behav Sci (Basel). 2026 Oct 9;16(10):1852. doi: 10.3390/bs16101852 (PMC13701205; doi:10.3390/bs16101852)
Supplement: Supplementary file 1 [file behavsci-16-01852-s001.zip › behavsci-4537863-supplementary.pdf]

Supplementary Syntax S1. Mplus Syntax for the Covariate-Adjusted Manual BCH Analysis of Cyberbullying Perpetration and Cybervictimization

```
TITLE:
  Covariate-adjusted manual BCH analysis of cyberbullying perpetration;

DATA:
  FILE = "bch_weights.dat";
  FORMAT = FREE;
  LISTWISE = ON;

VARIABLE:
  NAMES = ZTRUST
    ZCOM
    ZALI
    AGE
    GENDER
    FEDU
    MEDU
    CBPMEAN
    CVMEAN
    BCHW1
    BCHW2
    BCHW3
    BCHW4;

  USEVARIABLES = AGE
    GENDER
    FEDU
    MEDU
    CBPMEAN
    BCHW1
    BCHW2
    BCHW3
    BCHW4;

  MISSING = all(999999);

  CLASSES = c1(4);
  TRAINING = bchw1-bchw4(BCH);

DEFINE:
  CENTER AGE GENDER FEDU MEDU (GRANDMEAN);
```

```
ANALYSIS:
  TYPE = MIXTURE;
  ESTIMATOR = MLR;
  STARTS = 0;
  PROCESSORS = 8;
MODEL:
  %OVERALL%

    c1 ON AGE
          GENDER
          FEDU
          MEDU;
    CBPMEAN ON AGE
               GENDER
               FEDU
               MEDU;
    CBPMEAN;

    %c1#1%
    [CBPMEAN] (m1);
    CBPMEAN (v1);

    %c1#2%
    [CBPMEAN] (m2);
    CBPMEAN (v2);

    %c1#3%
    [CBPMEAN] (m3);
    CBPMEAN (v3);

    %c1#4%
    [CBPMEAN] (m4);
    CBPMEAN (v4);

MODEL TEST:
    0 = m1 - m2;
    0 = m1 - m3;
    0 = m1 - m4;
OUTPUT:
  SAMPSTAT RESIDUAL TECH1 TECH3 CINTERVAL;
```

```
TITLE:
  Covariate-adjusted manual BCH analysis of cybervictimization;

DATA:
  FILE = "bch_weights.dat";
  FORMAT = FREE;
  LISTWISE = ON;

VARIABLE:
  NAMES = ZTRUST
    ZCOM
    ZALI
    AGE
    GENDER
    FEDU
    MEDU
    CBPMEAN
    CVMEAN
    BCHW1
    BCHW2
    BCHW3
    BCHW4;

  USEVARIABLES = AGE
    GENDER
    FEDU
    MEDU
    CVMEAN
    BCHW1
    BCHW2
    BCHW3
    BCHW4;

  MISSING = all(999999);

  CLASSES = c1(4);
  TRAINING = bchw1-bchw4(BCH);

DEFINE:
  CENTER AGE GENDER FEDU MEDU (GRANDMEAN);

ANALYSIS:
  TYPE = MIXTURE;
  ESTIMATOR = MLR;
```

```
   STARTS = 0;
   PROCESSORS = 8;
MODEL:
   %OVERALL%

      c1 ON AGE
              GENDER
              FEDU
              MEDU;
      CVMEAN ON AGE
                  GENDER
                  FEDU
                  MEDU;
      CVMEAN;

      %c1#1%
      [CVMEAN] (m1);
      CVMEAN (v1);

      %c1#2%
      [CVMEAN] (m2);
      CVMEAN (v2);

      %c1#3%
      [CVMEAN] (m3);
      CVMEAN (v3);

      %c1#4%
      [CVMEAN] (m4);
      CVMEAN (v4);

MODEL TEST:
      0 = m1 - m2;
      0 = m1 - m3;
      0 = m1 - m4;
OUTPUT:
   SAMPSTAT RESIDUAL TECH1 TECH3 CINTERVAL;
```
